# Incidence and predictors of Sudan ebolavirus transmission among contacts in Uganda in 2022: a retrospective cohort study

**DOI:** 10.1186/s12889-025-24739-0

**Published:** 2025-11-05

**Authors:** Muhamed Mulongo, Joseph K. B. Matovu, Yovani A. M. Lubaale, Peter Olupot-Olupot

**Affiliations:** 1 Department of Community and Public Health, Busitema University Faculty of Health Sciences, Mbale City, East Africa Uganda; 2 Makerere University School of Public Health, Kampala City, East Africa Uganda; 3 Mbale Clinical Research Institute, Mbale Regional Referral Hospital, Mbale City, East Africa Uganda

**Keywords:** Ebola transmission incidence, Predictors, Uganda, Sudan ebolavirus

## Abstract

**Background:**

Ebola disease (EBOD) is a highly lethal zoonotic viral hemorrhagic fever that is responsible for 41 outbreaks globally. Ebola transmission is a key driver of the magnitude and complexity of outbreaks, yet incidence rate during outbreaks is not fully studied. This study aimed to determine the incidence and predictors of Sudan Ebolavirus transmission during the 2022 outbreak in Uganda.

**Methods:**

We conducted a retrospective cohort study leveraging surveillance records for individuals identified as contacts, and collected between September and December 2022 during the Sudan Ebolavirus Disease (SUVD) outbreak in Uganda. Data were extracted and analyzed via Stata/SE 15. This analysis involved calculating incidence rates and assessing predictors of Ebola transmission through appropriate multivariable regression models, and controlling for potential confounders like sex, age, health worker, and place of contact. The study protocol was approved by the Busitema University Research Ethics Committee (BUFHS-2023-145). Individual informed consent was waived, and all the data were anonymized.

**Results:**

3140 contacts were included in the study, with 50.6% being female and a mean age of 24 years. The incidence rate of SUVD was 1.6 cases per 1,000 person-days of follow-up. Kaplan Meier survival function showed that the risk for being diagnosed with SUVD extended beyond 21 days, up to the 28th day postexposure. The incidence of SUVD in secondary transmission settings was 1.8 times higher than that in primary transmission settings (IRR = 1.81, 95% CI = 1.202–2.735; *P* < 0.001). After controlling for sex, age, health worker, and place of contact, the significant predictors of SUVD transmission were high-risk contact status (aHR = 2.5, 95% CI: 1.68–3.72; *P* < 0.001) and male sex in secondary transmission settings (aHR = 2.14, 95% CI = 1.15–4.01; *P* = 0.02).

**Conclusions:**

This study revealed a high incidence of Ebola among contacts, with cases emerging beyond the standard 21-day follow-up period. The incidence was notably higher in secondary transmission settings, with high-risk contacts and males being particularly vulnerable. These findings suggest the need to revise contact tracing protocols (extend beyond 21 days), and prioritize follow-up on the basis of risk stratification. Further research is warranted to explore sex-related differences in secondary transmission dynamics.

## Background

Emerging and re-emerging infectious diseases are major public health challenges in the world, majority of which, are zoonotic in origin. Ebola disease (EBOD) is a highly fatal emerging and re-emerging zoonotic disease that is a major threat to global health. It is caused by a virus belonging to the family *Filoviridae* and genus *Ebolavirus*. The *Ebolavirus* genus has six species, which are *Ebola* (*Zaire) virus* (EBOV), *Sudan virus* (SUDV), *Bundibugyo virus* (BDBV), *Tai Forest virus* (TAFV), *Reston virus* (RESTV), and *Bombali virus* [1, 2]. The first four species cause disease in humans and the International Classification of Diseases (ICD) 11th Revision recognizes all diseases caused by viruses belonging to the genus *Ebolavirus* as Ebola Disease (EBOD). The diseases caused by the specific species of the *Ebolavirus* are BDVD caused by *Bundibugyo Ebolavirus*; SUVD caused by *Sudan Ebolavirus*, EVD caused by *Ebola (Zaire) Ebolavirus* and; TAFVD caused by the *Tai Forest Ebolavirus* [2, 3].

Since its discovery in 1976, *Ebolavirus* has caused 41 outbreaks globally, 33 of which have occurred in Africa [4]. The largest outbreak occurred in West Africa in 2014, where 28,610 cases were registered, and the case fatality rate (CFR) was 40% [5]. Uganda has experienced six outbreaks since 2000, four of which were caused by *Sudan Ebolavirus* (SUDV) species [4, 6]. The latest outbreak in Uganda occurred in 2022, caused by SUDV with 164 confirmed cases, and a CFR of 39% [7].

Ebola disease outbreaks begin when humans come into contact with the body fluids of infected wild animals, and index cases are usually hard to identify because the presentation in adults and children is similar to that in other common clinical conditions [8]. The incubation period is 2 to 21 days, and by the time an outbreak is confirmed and interventions are initiated, many people will be infected and die from the disease [9]. Secondary transmission, which refers to the spread of infection from a person who is confirmed to have the infection to other people through direct or indirect exposure, determines the size of EBOD outbreaks and case fatality rates, which range from 25 to 90% depending on the context and nature of the response [10]. The degree of transmission in these outbreaks varies. In some outbreaks, transmission stopped in the index case or after a few secondary cases. If transmission is not well understood and controlled through timely and effective interventions, outbreaks can rapidly expand to become global public health problems [11].

The current understanding of EBOD transmission was developed on the basis of epidemiological data from previous outbreaks and animal models [12–15]. Observations, lessons, and experiences from previous outbreaks have also been used to develop effective interventions for predicting, preparing, and responding effectively, in order to prevent small outbreaks from growing into large epidemics [16]. Few prospective studies have been conducted during outbreaks as part of public health interventions, and some studies have been conducted with surviving patients and contacts after the outbreaks [17].

Ebola transmission during outbreaks has not been reported uniformly, which limits comparisons and a detailed understanding of Ebola spread. Previous studies on Ebola transmission used secondary attack rates (SARs) instead of incidence rates [18]. Other studies also reported reproductive numbers, and their findings vary [19–21]. Most studies used cumulative numbers and descriptive statistics to estimate transmission [18]. Studies conducted from previous outbreaks also show that exposure to body fluids of an infected person is the highest risk for Ebola transmission but the exposure levels also vary with sociodemographic and other factors [22–24].

Ebola transmission has not been comprehensively analyzed for all outbreaks [18, 24], yet outbreaks are usually unique because of the causative agent, context, and timing of interventions, which may result in varying sizes of outbreaks, rates of spread, and CFRs [25, 26]. This study aimed to determine the incidence and predictors of Ebola transmission during the 2022 outbreak. The study hypothesized no difference in Ebola transmission rates between primary and secondary transmission settings.

## Methods

### Study design

A retrospective cohort study was conducted in April 2024 using surveillance records collected between September and December 2022 during the SUVD outbreak in Uganda.

### Study site and setting

The study was conducted at the district headquarters in Mubende and Kassanda districts, Central Uganda. Mubende and Kassanda districts were selected purposively because they were the epicenters of the SUVD outbreak in 2022. These two districts registered 80% of cases (Mubende 45% & Kassanda 35%), and 68% of the contacts (Mubende 46% and Kassanda 22%) during the outbreak [27]. Mubende district is located 160 km west of Kampala capital city along Kampala‒Fort Portal Road. Kassanda district neighbors Mubende in the south, and is located approximately 120 km west of the capital city of Kampala.

### Ebola disease outbreak and response in Uganda in 2022

Uganda’s Ministry of Health declared an outbreak of SUVD on 20th September 2022. The causative agent for this outbreak, confirmed by the Uganda Virus Research Institute (UVRI) via polymerase chain reaction (PCR), was *Sudan Ebolavirus* (SUDV). This outbreak started in Mubende district and spread to eight other districts. A total of 164 cases were registered during this outbreak, 55 of which died [7]. During the outbreak, surveillance teams comprising trained epidemiologists from the Ministry of Health (MoH), World Health Organization (WHO), partner agencies, and district health workers conducted case investigations for all confirmed cases. They traced and interviewed all the persons who had had contact (directly or indirectly) with a confirmed SUVD case to obtain their sociodemographic and exposure information. The information gathered per individual was recorded using standard forms for integrated disease surveillance and response (IDSR), recommended by the WHO.

All contacts were followed up in the same way irrespective of the type of exposure and other characteristics. Contacts were monitored daily by contact tracers and village health teams (VHTs), and their status was updated daily using contact tracing forms. Contacts who developed symptoms that met the case definition of a suspected case of SUVD were evacuated from the community using ambulances and taken to the isolation facility in Mubende district, where blood samples were drawn and *Ebolavirus* PCR tests were performed. The PCR results were entered into the Ministry of Health’s online results dispatch system (RDS), which is accessible only by authorized personnel. Information on contacts and cases was kept under control to ensure the confidentiality and safety of the records.

### Study population

The study included all individuals (children and adults) who were registered and followed up as contacts in Mubende and Kassanda districts during the 2022 SUVD outbreak. The study excluded contacts who were registered and followed up in other districts of Uganda and contacts with incomplete and unverifiable records.

### Measurement of variables

The outcome of interest was “SUVD diagnosis”, which was defined as an event in which a contact developed SUVD symptoms as per the case definition and tested positive for SUVD, confirmed by a positive *Ebolavirus* RNA polymerase chain reaction (PCR). This was a categorical variable with values of “Case” for a contact who developed SUVD, “Completed” for a contact who did not develop SUVD after follow-up, and “incomplete” for a contact who did not complete follow-up due to loss to follow-up. In survival analysis, the failure event was “a contact becoming a case of SUVD”. The time-to-event variable was the number of days calculated from two variables, i.e., “date of last contact” and “date of follow-up outcome”.

The independent variables were the sociodemographic and exposure characteristics of the contacts. Age (completed years) was a quantitative variable that was converted into a categorical variable using the following age groups: less than 5 years, 6–9 years, 10–19 years, 20–29 years, 30–39 years, 40–49 years and 50 years and above. Sex was a categorical variable defined as male or female. The relationship of the index case to contact was a categorical variable defined as the spouse, biological child, biological parent, neighbor, workmate, or other relationship. This variable was converted to “place of contact” such that the values were reduced to family, community, health care, school, and religious values.

The type of contact was a categorical variable defined as (1) sleeping in the same household with a confirmed case; (2) direct physical contact with the confirmed case (dead or alive); (3) touching his/her linens or body fluids; (4) eating or touching a sick or dead animal; and (5) other contact with contacts who were not in the categories above but were listed because they were close to a confirmed case but were not sure if they had contact with the body fluids of the infected person. Some contacts reported more than one type of exposure. This variable was recategorized on the basis of risk. Contacts with a combination that contained at least 2, 3, or 4 exposures were categorized as high risk. Contacts with exposure 1 were categorized as moderate risk, and contacts with only exposure 5 were categorized as low risk.

### Data extraction and analysis

Surveillance records collected during the 2022 SUVD outbreak were archived at the respective District Health Offices in the Mubende and Kassanda districts. The records were accessed by the research team after obtaining ethical approval from the Busitema University Research Ethics Committee (REC), and permission from the Chief Administrative Officers in the respective districts. Data for the study were extracted from the surveillance records using the data extraction tool developed by the researcher. The records were extracted anonymously to ensure the confidentiality of the study participants. The data were analyzed using STATA version 15 (StataCorp LLC, College Station, Texas, USA). Participant characteristics were summarized using frequencies, means, medians, and standard deviations.

The incidence and risk factors were determined using survival, bivariate, and multivariate analyses. The person-time contributed by each participant was the number of days from the last day of contact with a confirmed SUVD case to either diagnosis, loss-to-follow-up, death, or completion of follow-up. The study hypothesis was tested by comparing the Ebola transmission rate between primary and secondary transmission settings. The primary transmission setting was defined as the district where the outbreak started, with most of the cases being probable and without an epidemiologic link to index cases, as described by other scholars [28]. The primary transmission setting in this study was defined as the Mubende district because it is where the outbreak started in 2022, and many of the cases in this district did not have traceable epidemiologic links to confirmed or probable cases. Kassanda and other districts were considered secondary transmission settings because they are where the outbreak spread after Mubende district.

Kaplan‒Meier survival curves were used to show changes in the probability of remaining Ebola-free over time. The Cox proportional hazard regression model was used to conduct multivariable analysis to assess the predictors for Ebola transmission, and the results were reported using adjusted hazard ratios (aHRs). To test the study hypothesis, we compared the incidence of SUVD in primary and secondary transmission settings. Statistical significance was determined via 95% confidence intervals (CIs) and P-values less than 0.05. Contacts with missing data were excluded from the analysis at each stage.

## Results

### Characteristics of the study participants

**Fig. 1:**
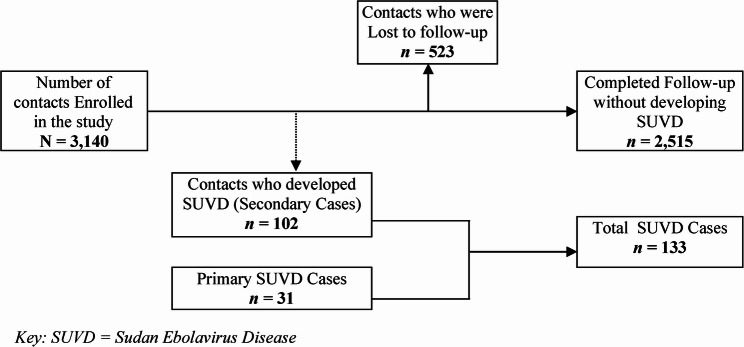
Flow chart showing participant enrollment, follow-up, and outcome

Figure 1 shows the participant enrollment, follow-up, and outcome data. A total of 3140 SUVD contacts were enrolled. 16% (*n* = 523) of the contacts were lost to follow-up, 3.2% (*n* = 102) developed SUVD, and 80.1% (*n* = 2515) ended the follow-up without developing SUVD.

Table 1 shows the background characteristics of the study participants. The mean age of the contacts was 24 years. 51% (*n* = 1590) of them were female. 40% were community contacts, 27.2% were family, and 13.8% were health facility contacts. 11% (*n* = 340) of the contacts were health workers. 51% were categorized by the surveillance team as moderate-risk contacts and 38% were categorized as high-risk contacts. 62% of the contacts were registered and followed up in Mubende district, and 36.6% were registered and followed up in Kassanda district. The median number of incubation days at the time of identification of the contacts was 5.5 days (interquartile range [IQR] = 8 days), and the median duration of follow-up of contacts was 21 (IQR = 2).

**Table 1:** Characteristics of SUVD contacts enrolled in the study

Characteristic	Did Contact developed SUVD		Total
	Yes (*n* = 102)	No (*n* = 3038)	*N* = 3140
	*n*(%)	*n*(%)	*n*(%)
Age, mean (SD*)	24.73 (14.5)	24.00 (15.6)	24.03 (15.6)
< 5 years	10 (9.8)	307 (10.1)	317 (10.1)
5–9 years	8 (7.8)	316 (10.4)	324 (10.3)
10–19 years	16 (15.7)	608 (20.0)	624 (19.9)
20–29 years	34 (33.3)	781 (25.7)	815 (26.0)
30–39 years	19 (18.6)	526 (17.3)	545 (17.4)
40–49 years	10 (9.8)	274 (9.0)	284 (9.0)
> 50 years	5 (4.9)	226 (7.4)	231 (7.4)
Sex			
Female	44 (43.1)	1546 (50.9)	1590 (50.6)
Male	58 (56.9)	1492 (49.1)	1550 (49.4)
Place of contact/exposure			
Community	49 (48.0)	1214 (40.0)	1263 (40.2)
Family	30 (29.4)	824 (27.1)	854 (27.2)
Health worker	7 (6.9)	428 (14.1)	435 (13.9)
Religious	1 (1.0)	19 (0.6)	20 (0.6)
School	3 (2.9)	156 (5.1)	159 (5.1)
Missing	12 (11.8)	397 (13.1)	409 (13.0)
Type of contact (risk level)			
High risk	64 (62.8)	1128 (37.1)	1192 (38.0)
Moderate risk	29 (28.4)	1568 (51.6)	1597 (50.9)
Low risk	5 (4.9)	304 (10.0)	309 (9.8)
Missing	4 (3.9)	38 (1.3)	42 (1.3)
Contact’s district of exposure			
Kassanda	55 (53.9)	1095 (36.0)	1150 (36.6)
Mubende	45 (44.1)	1911 (62.9)	1956 (62.3)
Others	2 (2.0)	32 (1.1)	34 (1.1)
Is contact a health worker?			
Yes	9 (8.8)	331 (10.9)	340 (10.8)
No	93 (91.2)	2707 (89.1)	2800 (89.2)
Number of days from date of last contact to end of follow up (person days of follow up)			
Mean (SD)	14.31 (7.0)	20.50 (5.4)	20.30 (5.5)
Median	15	21	21
Interquartile Range (Q†)	9 (11–20)	2 (20–22)	2 (20–22)
Range (Min–Max)	27 (1–28)	46 (1–47)	46 (1–47)

* SD Standard Deviation, *†Q* Quartile, *Min* minimum, *Max* maximum, *SUVD* Sudan Ebolavirus Disease

### Incidence of SUVD among contacts in central Uganda in 2022

Among the 3140 contacts who were exposed, 523 (16.7%) did not complete follow-up to ascertain their outcomes. These contacts were censored, and their number was reduced to half in the denominator. There were 102 contacts who developed SUVD, i.e., developed symptoms and had a positive PCR test. Therefore, the cumulative incidence of SUVD among contacts was 3.7% (Fig. 1). The study dataset was declared to be single-record survival-time data via Stata 15 software (Table 2). The time-to-event per person was calculated as the number of days from the person’s last date of contact with a confirmed case to the date of the outcome.

**Table 2:** Survival-time data and calculation of the incidence rate of SUVD

Parameter	Results		
Total participants in the study	3140		
Number of failure events	102		
Total person-time at risk	63,744 days		
SUVD Incidence rate	1.6 per 1000 person-days of follow up		
Survival time to diagnosis	25% (1st Quartile)	50% (Median)	75% (3rd Quartile)
	11	15	20

The total time at risk for the 3140 contacts in the study was 63,744 person-days. The incidence rate (IR) for SUVD was 1.6 cases per 1000 person-days of follow-up. The median period from the last date of exposure to the date of being diagnosed with SUVD was 15 days (interquartile range [IQR] = 9).

**Fig. 2:**
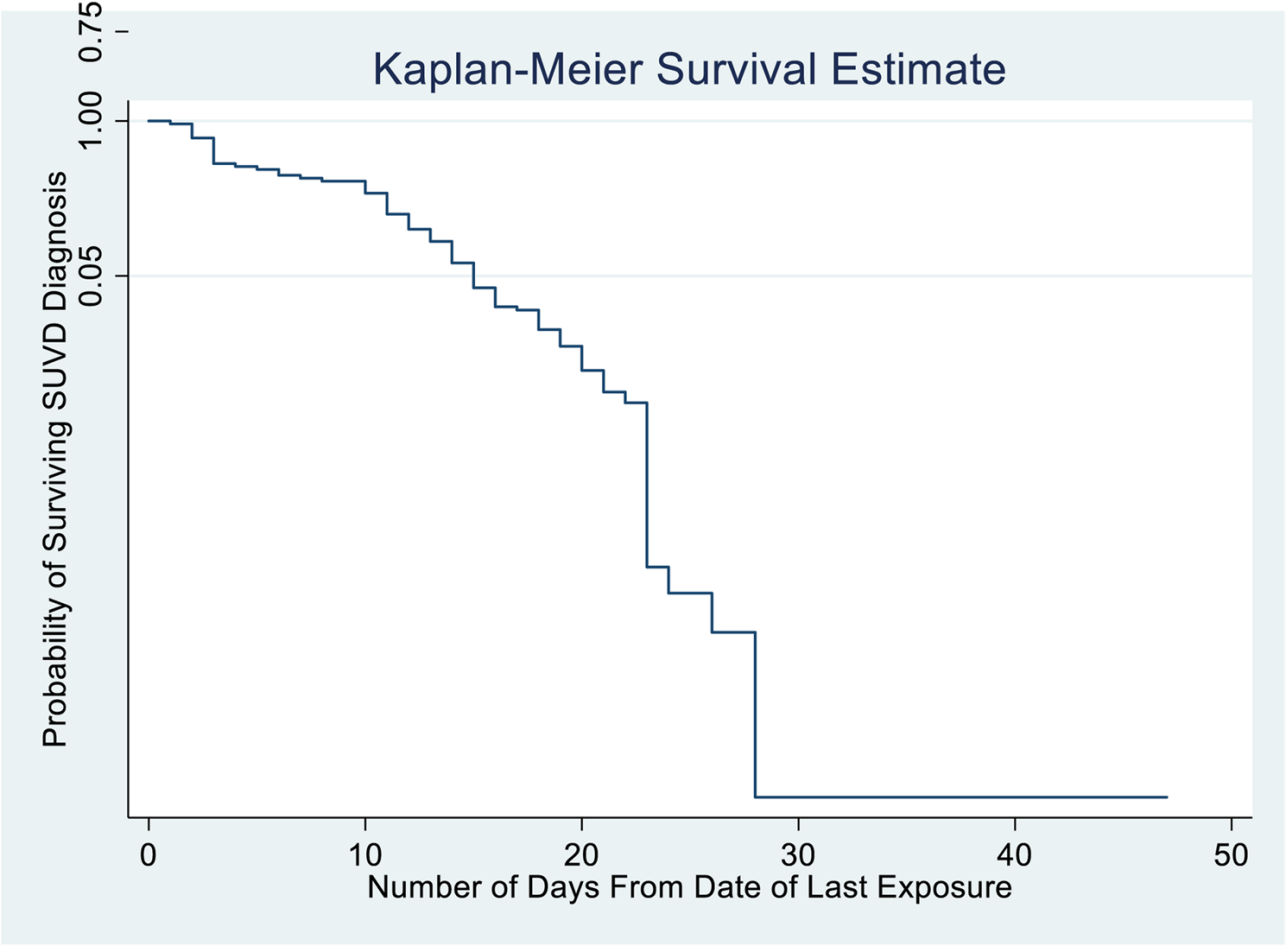
Kaplan Meier survival curve for the diagnosis of SUVD among contacts

The Kaplan–Meier survival function was used to determine the probabilities of remaining Ebola-free (surviving SUVD diagnosis) during follow-up (Fig. 2). The graph shows that the probability of remaining SUVD-free among contacts decreased mildly between the 1 st and 10th days of follow-up, decreased moderately between the 10th and 23rd days, and decreased significantly between the 23rd and 28th days. The risk of developing EBOD continued to the 28th day after exposure.

### Testing the study hypothesis

The incidence of SUVD in the secondary transmission setting was 1.8 times the incidence of SUVD in the primary transmission setting (IRR = 1.81, 95% CI = 1.202–2.735; *P* < 0.001). The secondary transmission setting had a 45% excess incidence of SUVD. Therefore, the null hypothesis is rejected (*P* < 0.001) (Table 3).

**Table 3:** Comparing SUVD incidence rates in primary and secondary transmission settings

	Secondary transmission setting (Exposed)	Primary transmission setting (Unexposed)	Total
SUVD cases	57	45	102
Time at risk	26,260	37,484	63,744
Incidence rate	0.00217	0.00120	0.0016

Incidence rate ratio = 1.81 (95%CI: 1.20–2.73, *P* < 0.001)

Attributable fraction in the exposed = 0.45 (95%CI: 0.17–0.63)

*SUVD* Ebola Sudan Virus Disease, *CI* Confidence Interval

### Bivariate analysis of risk factors for SUVD transmission among contacts

Bivariate analysis was conducted for the different sociodemographic and exposure characteristics to determine risk factors for SUVD incidence among contacts. Table 4 shows that the significant factors associated with the transmission of SUVD were the age group 20–29 years (incidence risk ratio [IRR] = 1.48; 95% CI: 0.95–2.26; *P* = 0.03), contacts in secondary transmission settings (IRR = 1.81; 95% CI: 1.20–2.73; *P* < 0.001), and high-risk contacts (IRR = 3.06; 95% CI: 1.99–4.79; *P* < 0.001). The contacts who were exposed to health facilities had a low risk of developing SUVD (IRR = 0.5 95% CI: 0.20–1.08; *P* = 0.03).

**Table 4:** Bivariate analysis of risk factors for transmission of SUVD among contacts

Risk Factors	Total (*N*)	SUVD Cases (*n*/%)	IR (per 1000)	IRR	Confidence Intervals	*P* value
Sex						
Female	1590	44 (2.77)	1.37	1		
Male	1550	58 (3.74)	1.83	1.33	0.89–2.02	0.07
Age categories						
≤ 5years	317	10 (3.15)	1.48	1.21	0.49–2.83	0.31
6-9years	324	8 (2.47)	1.14	0.93	0.35–2.31	0.45
10-19years	624	16 (2.56)	1.23	1		
20-29years	815	34 (4.17)	2.11	1.72	0.93–3.34	0.03
30-39years	545	19 (3.49)	1.77	1.45	0.70–3.01	0.14
40-49years	284	10 (3.52)	1.81	1.48	0.60–4.47	0.17
≥ 50years	231	5 (2.16)	1.08	0.88	0.23–2.52	0.42
Type of risk						
Low risk	309	5 (1.62)	0.78	1		
Moderate risk	1597	29 (1.82)	0.89	1.16	0.44–3.79	0.41
High risk	1192	64 (5.37)	2.67	3.44	1.40–10.95	< 0.001
Place of contact						
Family	854	30 (3.51)	1.69	1		
Community	1263	49 (3.88)	1.88	1.12	0.69–1.82	0.32
Health facility	435	7 (1.61)	0.88	0.52	0.19–1.21	0.05
Religious	20	1 (5.00)	2.41	1.43	0.04–8.59	0.33
School	159	3 (1.89)	0.89	0.53	0.10–1.71	0.15
Type of transmission setting						
Primary	1956	45 (2.30)	1.20	1		
Secondary setting	1150	57 (4.78)	2.17	1.78	1.18–2.72	< 0.001
Health workers						
Non-Health workers	2800	93 (3.32)	1.61	1		
Health workers	340	9 (2.65)	1.48	0.92	0.41–1.82	0.42

Key: *IR* Incidence Rate, *IRR* Incidence Rate Ratio

### Multivariable analysis for risk factors for SUVD secondary transmission

The risk factors included in the multivariable analysis were age, sex, type of risk, place of contact, health worker status, and transmission settings on the basis of their significant association in the bivariate analysis or potential confounding. The risk factors fit into the Cox proportional hazard model (Table 5).

**Table 5:** Cox hazards regression analysis for SUVD risk factors stratified by sex

Risk Factor	Male			Female		
	aHR	95% CI	*P* value	aHR	95% CI	*P* value
Age	1.01	0.99–1.03	0.23	1.01	0.99–1.03	0.44
Type of contact	3.26	1.86–5.69	< 0.001	1.96	1.12–3.42	0.02
Health workers	1.93	0.78–4.75	0.16	0.74	0.17–3.21	0.69
Place of contact	0.76	0.52–1.10	0.15	0.92	0.64–1.33	0.67
Transmission setting	2.14	1.15–4.01	0.02	1.01	0.51–2.00	0.98

Key: *aHR* Adjusted Hazard Ratio, *CI* Confidence Interval

Log rank test: Chi2 = 27.74; *P* < 0.001

The log-rank tests were conducted to test the fitness of the regression model, and the results showed that the model was adequate (Chi2 = 27.74; *P* < 0.001). There was a statistically significant risk of developing SUVD associated with the type of risk (aHR = 2.5, 95% CI: 1.68–3.72; *P* < 0.001) and male sex in secondary transmission settings (aHR = 2.14, 95% CI: 1.15–4.01; *P* = 0.02) after adjusting for the other risk factors in the regression model. There was no statistically significant risk of SUVD associated with age, sex, place of contact, transmission settings, or health workers after adjustment.

## Discussion

This study found that during the SUVD outbreak in Uganda in 2022, the cumulative incidence of SUVD among contacts was 3.67%, the incidence rate was 1.6 cases per 1000 person-days of follow-up, the probability of remaining free of Ebola virus significantly decreased between the 10th and 28th days of follow-up, the incidence in secondary transmission setting was almost twice that in the primary transmission setting, and the statistically significant risk factors for SUVD transmission was the type of contact, and male sex in secondary transmission settings.

### The incidence of SUVD among contacts in central Uganda in 2022

The cumulative incidence of SUVD among contacts during the 2022 outbreak in Uganda was 3.67%, indicating that almost 4 in 100 contacts developed SUVD. This is similar to the secondary attack rates (SARs) reported for household contacts in previous studies, which ranged from 2.5 to 27% [18]. A meta-analysis by Dean et al. (2016) revealed that SARs for SUDV outbreaks ranged between 11% and 27%. However, studies that combined community and household contacts had relatively lower SARs, similar to the cumulative incidence reported in this study.

The incidence rate of SUVD among contacts was 1.6 cases per 1000 person-days of follow-up, implying that approximately 2 people developed Ebola if 1000 people were exposed and followed up for one day. In 2015, Glynn J.R. constructed age-specific incidence rates for the outbreak in West Africa and reported that the incidence rates were between 5 and 180 cases per 100,000 people in the general population [29]. These findings are quite different from those of this study because of the denominators used. We report individual-based person-time of exposure, which is a more accurate measure of incidence [29, 30]. The observed IR highlights the need for robust surveillance and effective control measures during EBOD outbreaks to reduce reproductive numbers.

The Kaplan–Meier survival function revealed that the probability of remaining free of the Ebola virus significantly decreased between the 10th and 28th days of follow-up. These findings show that the risk of developing Ebola transmission continued from the time of exposure up to 28 days (four weeks). Previous studies have shown that contacts develop EBOD between 2 and 21 days after exposure [9, 31]. The extended period of incubation in this outbreak could have been due to delayed diagnosis of cases, repeated exposure, or a longer incubation period associated with the strain of the *Ebolavirus* during this outbreak, as reported previously [32]. The 28th day extended risk in the 2022 outbreak is not compared to previous outbreaks, like the 2014 outbreak in West Africa, because incidence rates and survival analysis were not described in those outbreaks. Guidelines for contact tracing recommend that follow-up is stopped after 21 days [33]. This implies that late cases that develop symptoms after 21 days can be missed, resulting in a propagated outbreak. These findings suggest the need to revise guidelines on the duration of contact follow-up during EBOD outbreaks, especially those caused by SUDV.

The incidence of SUVD in the secondary transmission setting was almost twice that in the primary transmission setting. This could be due to the rapid human-to-human spread of SUVD [1, 34]. These findings provide evidence for the importance of risk-based prioritization and interventions for the follow-up of contacts.

### Predictors of SUVD transmission among contacts

This study found that the statistically significant risk factors for SUVD transmission were the type of contact and male sex in secondary transmission settings. Contacts with a high risk of contact with body fluids of a confirmed case were developing SUVD, and males in Kassanda district had a high risk of developing SUVD. These findings indicate that high-risk exposure to SUVD may result in transmission irrespective of an individual’s sociodemographic characteristics. Migamba et al. also conducted a retrospective study of the same outbreak and reported that direct contact was associated with a high risk of SUVD (aRR = 1.9, 95% CI: 1.1–9.7) [35]. Although a small sample was used, the findings complement those of this study and add evidence to other studies that have shown that being exposed to the body fluids of an infected person poses a high risk of being infected with Ebola [23, 24]. However, Rowe et al. demonstrated that transmission may not occur in all cases of exposure [36]. Chowel and Nishiura argued that the context of an outbreak determines the transmission of Ebola, and they recommended research on spatiotemporal changes in factors such as behavior, contact networks, and hospital infection measures as predictors of transmission patterns of EBOD [37]. This implies that during outbreaks, people should be sensitized to avoid high-risk exposures, and there should be a prioritization of high-risk contacts with effective interventions to prevent transmission.

Being male in a secondary transmission setting was a risk factor for SUVD in this outbreak, which could have been due to the context and social factors during this outbreak. Some scholars, such as Robert & Edmund [38], did not find sex to be a key factor in the transmission of Ebola in Guinea in 2014. However, some scholars have demonstrated that demographic characteristics influence the spread of epidemics such as MERS and Ebola, but their studies did not identify specific risk differences between females and males [39]. The men in secondary transmission settings could have been at greater risk due to community and environmental factors [25, 26, 38, 40]. More studies should be conducted to understand the sex-related differences in transmission of Ebola.

### Study strengths and limitations

The incidence rate with person-time of observation is a more specific and effective measure of transmission rate than the secondary attack rates used in most studies. The size of this study is greater than the numbers used to study secondary transmission in previous studies [1, 18, 32]. However, the incidence rate of SUVD could have been underestimated due to the high index of suspicion, which could have resulted in medical surveillance bias, as described by Sizlo and Neito [30]. Using only PCR for diagnosis during the outbreak could have resulted in an underestimation of SUVD incidence due to missing early and late cases. There was potential misclassification in this study due to incomplete records.

## Conclusion

This study revealed a high incidence of SUVD among contacts, with new cases continuing to emerge up to the 28th day post-exposure. Compared with primary settings, secondary transmission settings posed nearly twice the risk of SUVD transmission. The key predictors of SUVD transmission included the type of exposure and male sex in secondary settings. These findings underscore important aspects of EBOD transmission dynamics, including the incidence among contacts, the potential for prolonged incubation periods, and the differential risks between primary and secondary transmission environments. On the basis of these insights, the following recommendations are proposed:

Further research should evaluate the necessity of extending the follow-up period for contacts during SUVD outbreaks from the current 21 days to 28 days post-exposure. Enhanced attention should be given to high-risk contacts and male contacts in secondary transmission settings, potentially through interventions such as institutional quarantine and strengthened infection prevention and control measures. The development of robust surveillance systems for the early detection of EBOD outbreaks is critical for mitigating the incidence of Ebola. Further investigations are needed to explore the observed sex differences in SUVD incidence, with the aim of tailoring more effective prevention strategies.

## Data Availability

The data that support the findings of this study are not openly available due to reasons of sensitivity and are available from the corresponding author upon reasonable request.

## Abbreviations

CDC: Centers for disease control and prevention
CFR: Case fatality rate
EBOD: Ebola disease
EHF: Ebola hemorrhagic fever
ELISA: Enzyme-linked immunosorbent assay
EVD: Ebola virus disease
ICD: International classification of disease
IDSR: Integrated disease surveillance and response
IPC: Infectious prevention and control
MoH: Ministry of health
PCR: Polymerase chain reaction
SAR: Secondary attack rate
SUDV: *Ebola Sudan virus*
SUVD: Ebola Sudan virus disease
VHT: Village health team
WHO: World health organization
